# Valproate-Induced Hyperammonemic Encephalopathy

**DOI:** 10.7759/cureus.1593

**Published:** 2017-08-22

**Authors:** Faiza Farooq, Javeria Sahib Din, Ali M Khan, Syeda Naqvi, Shanila Shagufta, Abdul Mohit

**Affiliations:** 1 Psychiatry, Kings County Hospital Center; 2 Jinnah Postgraduate Medical Centre, Jinnah Sindh Medical University (SMC); 3 New York Medical College, Kings County Hospital; 4 Behavioral Health, Kings County Hospital Center

**Keywords:** psychiatry, valproate, hepatotoxicity, carnitine, hyperammonium

## Abstract

Valproate is the best choice drug for a variety of medical conditions. As with any other drug, it has adverse effects, and it is important to emphasize the possibility of those adverse effects to prevent complications. We present the case history of a 44-year-old male with valproate-induced hyperammonemic encephalopathy, despite having normal liver function tests. This case includes a detailed literature review of this rare adverse event. In the light of this case report, we illustrate the importance of checking ammonia levels in all psychiatric patients receiving valproate as a treatment who present with new onset neurological symptoms or altered mental status.

## Introduction

Valproate is generally used as an anticonvulsant for the treatment of seizures. In the past several years, its use in bipolar disorders as a mood stabilizer and in psychotic disorders, such as schizophrenia and schizoaffective disorder, along with an antipsychotic has been shown to be successful [1].

Valproate is considered as safe with a wide reference range of 50 to 125. In rare instances, it can cause hyperammonemia, leading to hyperammonemic encephalopathy in the absence of hepatotoxicity or elevated liver function tests (LFTs). The majority of the case reports on valproate-associated hyperammonemic encephalopathy were from neurology, but it was psychiatry in which it was reported for the first time back in 1995 [2]. Here, in this case report, we are trying to emphasize the importance of keeping an eye on the blood ammonia levels while using valproate, especially in the presence of new onset neurological symptoms like tremor or altered mental status, despite normal EKG and LFTs.

## Case presentation

We present the case of 44-year-old Haitian-American male suffering from a schizoaffective disorder since 2005. The patient was born and raised by a single parent and immigrated to the United States. Since 2012, he has been admitted to the inpatient psychiatric unit approximately two to three times a year and generally remains on the unit for a period of three to four weeks each time.

In 2012, the patient was brought to the emergency department after reportedly agitated behavior in a restaurant and threatening customers where he had florid disorganization and psychosis. Baselines, including a toxicology screen, detailed urine report, and liver function test (LFT), were all normal. He was held on a legal status and treated with oral risperidone, 2 mg, and oral valproate, 500 mg.

After one month of treatment with valproate, the patient appeared cooperative, well-groomed, alert, awake, and oriented with a valproate level of 50. After six weeks, a decision was made to increase the dose of valproate. The patient's dose was increased to 750 mg of valproate. During the day, the patient was noted to have abnormal twitching movements of his head and appeared drowsy. Baseline labs, including a complete blood count (CBC), basic metabolic panel (BMP), liver function test (LFT), electrocardiography (ECG), and computed tomography of the brain (CT brain), were ordered and a neurology consult was taken. Shortly thereafter, the patient became unresponsive, help was immediately called, and the patient was transferred to the Intensive Care Unit for further management. His ammonia level was found to be 383, but no hepatotoxicity was seen in his blood studies (normal LFTs) and he had a normal EKG. The patient had received an oral dose of valproate in the morning, thus raising our concern for valproate-induced hyperammonemic encephalopathy. We discontinued the valproate and started treatment with phenytoin.

Blood was drawn to check the valproate level after the first week of increased dose, which was found to be 110; this was towards the higher side of normal but still was not high enough to cause hyperammonemic encephalopathy. Valproate was already stopped and the ammonia level dropped to 44 after two days with improvement in the patient’s clinical status. A CT scan was repeated after four weeks and it was completely normal.

## Discussion

Valproate is generally used in several neurological and psychiatric disorders. Although it is primarily used in mood disorders, it can be used in combination with antipsychotics in schizophrenia and schizoaffective disorders. It is reported that the broad spectrum of adverse effects of valproate range from dizziness, unsteadiness, headache, blurred vision, and nystagmus (as being the less common adverse effects) to tremor being the most common one affecting our central nervous system. Valproate-induced hepatotoxicity is a rare adverse event occurring in approximately 1 in 20,000 patients. Valproate-induced encephalopathy is also a well-known adverse event, developing in association with hyperammonemia. It is characterized by impaired cognition, drowsiness, and apathy. It occurs shortly after the commencement of valproate with a rapid and complete resolution of symptoms with regained consciousness after treatment with valproate has been discontinued. This is an idiosyncratic reaction which, by definition, means it is unpredictable and would not always be related to plasma levels or valproate dose, increased ammonia levels, or abnormal LFTs. Development of these well-recognized serious side effects with the use of valproate is mostly influenced by factors such as susceptibility of an individual to develop these side effects, underlying urea cycle disorders, and coadministration of drugs that alter the pharmacodynamics and pharmacokinetics of valproate [3-4].

As the valproate level was only towards the higher side of the normal range, there is the possibility of the inherent genetic polymorphism of the enzyme, carbamoyl phosphate synthase 1 of the urea cycle. This might have led to the reduced activity of this enzyme, augmenting the rise in ammonia level following the administration of valproate. However, co-administration of certain medications that have the property of inducing the enzymes (e.g., phenytoin, phenobarbital, and carbamazepine) has been shown to change the activity of carbamoyl phosphate synthase activity [5]. It was found in one case report that concomitant administration of risperidone and valproate can spike the ammonia level. Physicians should stay alerted and need to realize the importance of monitoring the ammonia level while treating the patient with these two drugs concomitantly, although more light should be shed in this regard with additional research to draw a conclusion [6].

There are several mechanisms proposed that could have possibly been causing hyperammonemia associated with the valproate use. Briefly, valproate is a protein-bound branched-chain fatty acid that is extensively metabolized in the liver, mainly by glucuronidation in the cytosol and beta-oxidation in the mitochondria via carnitine shuttle with only 10% being metabolized by omega-oxidation. Thus, when valproate is transported into the mitochondria in conjugation with carnitine for beta-oxidation, it interferes with the transportation of other fatty acids, which may result in attenuation of mitochondrial acetyl coenzyme A (acetyl-CoA) production via beta-oxidation of other fatty acids. Less acetyl-CoA production further leads to the decreased production of N-acetyl-glutamic acid as it is required for the allosteric activation of an enzyme of the urea cycle, carbamoyl-phosphate synthetase 1, thereby, disrupting the urea cycle and leading to a rise in ammonia levels. Another proposed mechanism is the metabolism of valproate via omega-oxidation leading to the formation of 4-en-valproic acid, a toxic metabolite, which results in further impairment of carbamoyl-phosphate synthetase function. When the carnitine shuttles are saturated by valproate, beta-oxidation decreases and there is a shift towards omega oxidation, leading to further build-up of the toxic metabolites, inhibiting the carbamoyl-phosphate synthetase function and attenuation of the urea cycle [7-8].

Hence, carnitine supplementation not only shifts the metabolism of valproate towards the beta-oxidation, resulting in less production of toxic metabolites via omega-oxidation, but also allows the patient to use valproate to control their symptoms. It is evidenced by a case report in which the ammonia level was brought down within the reference range with the supplementation of carnitine with the continuation of valproate. However, as this case report was limited to one patient only, more patients should be studied to come to a strong conclusion [8]. Guo, et al. has described a valproate-induced hyperammonemic encephalopathy after craniotomy, which again highlights the fact that valproate has serious adverse effects and should be discontinued when therapeutic results are achieved [9-10].

## Conclusions

In light of this case report, we can conclude that blood ammonia levels should be checked in patients who develop any new onset neurological symptoms while being treated with valproate as it can lead to hyperammonemic encephalopathy, even when the valproate level is in the reference range and liver function tests are normal. Given the fact that the valproate level was only towards the higher side of the reference range, further workup should also be considered in such cases to rule out a genetic deficiency of the urea cycle enzyme per se as it can contribute to the acuteness of the symptoms.

## References

[1] Victoroff J, Coburn K, Reeve A (2014). Pharmacological management of persistent hostility and aggression in persons with schizophrenia spectrum disorders: a systematic review. J Neuropsychiatry Clin Neurosci.

[2] Aiyer R, Seide M, Stern RG (2016). Valproic acid induced hyperammonemia in a long time treated patient. Case Rep Psychiatry.

[3] Kimmel RJ, Irwin SA, Meyer JM (2005). Valproic acid-associated hyperammonemic encephalopathy: a case report from the psychiatric setting. Int Clin Psychopharmacol.

[4] Caruana Galizia E, Isaacs JD, Cock HR (2017). Non-hyperammonaemic valproate encephalopathy after 20 years of treatment. Epilepsy Behav Case Rep.

[5] Ando M, Amayasu H, Itai T, Yoshida H (2017). Association between the blood concentrations of ammonia and carnitine/amino acid of schizophrenic patients treated with valproic acid. Biopsychosoc Med.

[6] Carlson T, Reynolds CA, Caplan R (2007). Case report: Valproic acid and risperidone treatment leading to development of hyperammonemia and mania. J Am Acad Child Adolesc Psychiatry.

[7] Mojumder DK, De Oleo RR (2014). Differential ammonia decay kinetics indicates more than one concurrent etiological mechanism for symptomatic hyperammonemia caused by valproate overdose. Indian J Pharmacol.

[8] Maldonado C, Guevara N, Silveira A (2017). L-Carnitine supplementation to reverse hyperammonemia in a patient undergoing chronic valproic acid treatment: A case report. J Int Med Res.

[9] Guo X, Wei J, Gao L (2017). Hyperammonemic coma after craniotomy: Hepatic encephalopathy from upper gastrointestinal hemorrhage or valproate side effect?. Medicine (Baltimore).

[10] Dixit S, Namdeo M, Azad S (2015). Valproate induced delirium due to hyperammonemia in a case of acute mania: a diagnostic dilemma. J Clin Diagn Res.

